# Venetoclax-Based Regimens for Relapsed/Refractory Acute Myeloid Leukemia in a Real-Life Setting: A Retrospective Single-Center Experience

**DOI:** 10.3390/jcm10081684

**Published:** 2021-04-14

**Authors:** Matteo Piccini, Sofia Pilerci, Marta Merlini, Pietro Grieco, Barbara Scappini, Sara Bencini, Benedetta Peruzzi, Roberto Caporale, Leonardo Signori, Fabiana Pancani, Alessandro Maria Vannucchi, Giacomo Gianfaldoni

**Affiliations:** 1 SOD Ematologia, Università degli Studi di Firenze e Azienda Ospedaliera Universitaria Careggi, 50139 Firenze, Italy; piccinim@aou-careggi.toscana.it (M.P.); scappinib@aou-careggi.toscana.it (B.S.); a.vannucchi@unifi.it (A.M.V.); 2 Scuola di Specializzazione in Ematologia, Università degli Studi di Firenze, 50139 Firenze, Italy; sofipilerci@gmail.com (S.P.); martamerlini1992@gmail.com (M.M.); 3 SOD Ematologia, Ospedale San Donato, Azienda Usl Toscana Sud-Est, 20121 Milano, Italy; p.grieco89@gmail.com; 4 SOD Centro Diagnostico di Citofluorimetria e Immunoterapia, Azienda Ospedaliera Universitaria Careggi, 50139 Firenze, Italy; bencinis@aou-careggi.toscana.it (S.B.); peruzzib@aou-careggi.toscana.it (B.P.); caporaler@aou-careggi.toscana.it (R.C.); 5 Centro di Ricerca e Innovazione per le Malattie Mieloproliferative (CRIMM), Università degli Studi e Azienda Ospedaliera Universitaria Careggi, 50139 Firenze, Italy; leonardo.signori@unifi.it (L.S.); fabiana.pancani@unifi.it (F.P.)

**Keywords:** leukemia, venetoclax, chemoresistant

## Abstract

Relapsed/refractory (R/R) acute myeloid leukemia (AML) is a largely unmet medical need, owing to the lack of standardized, effective treatment approaches, resulting in an overall dismal outcome. The only curative option for R/R AML patients is allogeneic hematopoietic stem cell transplantation (HSCT) which is only applicable in a fraction of patients due to the scarce efficacy and high toxicity of salvage regimens. Recently, a number of targeted agents with relatively favorable toxicity profiles have been explored in clinical trials for R/R AML patients. The Bcl-2 inhibitor venetoclax, in combination with hypomethylating agents or low dose cytarabine, has produced impressive results for newly diagnosed AML, while its role in R/R disease is not well defined yet. We retrospectively analyzed the clinical outcomes of 47 R/R AML patients treated with venetoclax-based regimens between March 2018 and December 2020 at our institution. Overall, we report a composite complete response rate of 55% with an overall acceptable toxicity profile. Outcomes were particularly favorable for NPM1 mutated patients, unlike for FLT3-ITD positive patients irrespective of NPM1 status. For patients treated with intention to transplant, the procedure could be finally performed in 54%. These findings suggest a role for venetoclax-based regimens in R/R AML patients and support the design of prospective studies.

## 1. Introduction

Relapsed/refractory (R/R) acute myeloid leukemia (AML) has a largely unmet medical need because standardized, effective treatment approaches are lacking and the overall outcome is dismal. The only curative option for R/R AML patients is allogeneic hematopoietic stem cell transplantation (HSCT) [1], which unfortunately can only be exploited in a minority of the patients due to the poor efficacy and high toxicity of conventional induction regimens used to induce leukemia remission as bridge-to-transplant; in fact, a significant proportion of R/R AML patients are finally considered ineligible for HSCT due to persistent leukemia, advanced age and comorbidities [2]. Recently, a number of targeted agents with relatively favorable toxicity profiles have been explored in phase II and III trials for molecularly-defined subsets of R/R AML patients. Quizartinib and gilteritinib were tested in phase III studies in R/R patients with FLT3-mutated AML [3,4], resulting in gilteritinib to be approved by the FDA and other regulatory entities [5]. Additionally, ivosidenib and enasidenib entered phase I and II trials for IDH1 and IDH2-mutated R/R AML [6,7] ultimately resulting in their approval [8,9]. Venetoclax (VEN), a first-in-class, highly selective oral Bcl-2 inhibitor, was evaluated in newly diagnosed elderly/unfit patients in association with hypomethylating agents (HMA) [9] or low-dose cytarabine (LDAC) [10] yielding unprecedented complete remission (CR) rates and survival and, since its approval by FDA and EMA, has steadily become the standard of care in this patient subset. Conversely, the role of VEN in R/R AML has not been fully explored yet. Recently, a number of small retrospective studies evaluated the outcomes of R/R AML patients treated with VEN as single agent or in combination with other conventional agents, reporting overall encouraging results [11,12,13,14,15]. However, patient populations, treatment regimens and clinical outcomes varied among these studies. Here we report the clinical characteristics, treatment details, safety profile and clinical outcomes of 47 consecutive patients with R/R AML who were treated at our institution with VEN-based regimens.

## 2. Patients and Methods

### 2.1. Patients

We retrospectively analyzed outcomes of adult patients (>18 years old) with AML in the setting of relapse (first relapse, subsequent relapse or any post-HSCT relapse) or primary induction failure (PIF) who were treated consecutively at our institution between March 2018 and December 2020 with VEN in combination with azacitidine (AZA), decitabine (DAC) or LDAC. Relapse was defined as >5% blast on a bone marrow (BM) aspirate after a CR had been documented. Extramedullary relapse was defined as the presence of histologically confirmed blast cell infiltration outside the BM in a patient who had previously achieved a CR. PIF was defined as disease persistence in BM (>5% blasts) or extramedullary sites after a standard induction cycle (i.e.,: 3 + 7) and a high-dose cytarabine (HiDAC)-based salvage regimen or after a single intensified, HiDAC-containing, induction cycle. Response to conventional treatments and VEN-based regimens was adjudicated according to the European LeukemiaNet (ELN) 2017 recommendations [16]. In order to be eligible for inclusion into the study cohort, all patients must have received at least one full treatment cycle with at least a single evaluable response assessment. This study was approved by the local Ethical Committee, in accordance with the Declaration of Helsinki. 

### 2.2. Diagnostic Procedures

Diagnostic procedures at AML presentation were carried out according to ELN 2017 recommendations and this was considered a mandatory requirement for inclusion into the study cohort. Specifically, mandatory diagnostic procedures included morphological examination of peripheral blood and bone marrow smears (or trephine biopsy slides in case of dry tap), immunophenotyping, conventional karyotyping and mutation assessment for at least NPM1, FLT3 and CEBPA variants [17,18,19]. Analysis of FLT3 mutational status included variant allelic *ratio* (AR) assessment in case of internal tandem duplication (ITD) [20]. All patients and their siblings were HLA-typed at the time of diagnosis in order to facilitate referral to HSCT, if eventually indicated. Diagnostic procedures at the time of relapse or when refractoriness was ascertained included morphological examination of BM and PB smears, immunophenotyping, conventional karyotyping (for relapsing patients) and, where applicable, quantitative NPM1 mutation analysis and reassessment of FLT3 mutational status (including AR in ITD-positive cases at diagnosis). For selected patients, we also evaluated the magnitude of peripheral blast clearance (PBC) from baseline to day 4 of the first VEN cycle. PBC was defined as: $Log\;\frac{Absolute\;blast\;count\;baseline}{Absolute\;blast\;count\;day\;4}$; absolute blast counts were derived by multiplying absolute leukocyte count by the blast percentage as evaluated by microscopic examination of PB smears. 

Minimal residual disease (MRD) evaluation was carried out for all patients achieving a CR, including patients with incomplete peripheral counts recovery. MRD was measured at first CR achievement and as clinically indicated thereafter. In the case of NPM1 mutated patients, MRD evaluation was carried out by quantitative real-time polymerase chain reaction (RT-qPCR) using the Qiagen Ipsogen MutaQuant A kit (maximum sensitivity: 10^−6^). In the case of positivity for CBFB/MYH11 and RUNX1/RUNX1T1 fusion transcripts, MRD monitoring was carried out using the Qiagen Ipsogen CBFB/MYH11 A and RUNX1/RUNX1T1 kits (sensitivity: 10^−5^). In the case of patients lacking a suitable molecular marker, MRD monitoring was carried out by flow cytometry using a leukemia-associated immunophenotypic profile (LAIP)-based approach. Briefly, patient-specific immunophenotypic probes were designed based on baseline immunophenotypic profiles. Practically, two sets of 6–8 antigens were used at each MRD time-point in order to minimize the risk for false negative results caused by phenotypic shifts. Flow cytometry MRD positivity was defined according to the most recent ELN recommendations. 

### 2.3. Treatments

Venetoclax-based regimens were administered as follows: VEN was administered at the dose of 400 mg/d with a 3-day ramp-up on cycle 1 (100–200–400 mg). Initially, VEN was administered in a continuous schedule in 28-day cycles (dose level 1); due to growing evidence of excessive treatment-related myelosuppression, and according to recommendations from experts [21], we reduced the VEN exposure window to 21 days during the first cycle and 21 or 14 days during subsequent cycles (dose level 2). In case of profound cytopenias occurring despite the shortened VEN schedule, we allowed further reductions to 10 or even 7 days of treatment (dose level 3). A BM aspirate was performed to rule out disease persistence before any dose reduction in the individual patient. The off-label use of VEN was nominally authorized by the Agenza Italiana del Farmaco (AIFA) “Fondo 5%”. AZA was administered at 75 mg/mq/d subcutaneously with a 7-day (inpatients) or a 5 + 2 (outpatients) schedule. DAC was administered at 20 mg/mq/d intravenously for five consecutive days. Both AZA and DAC were given in 28 day cycles; however, cycles beyond the first one could be variably delayed in order to allow for peripheral counts recovery. LDAC was administered at 20 mg/mq/d subcutaneously for 7 or 10 days; LDAC cycles were repeated at 28-day intervals or as clinically indicated. Azole (posaconazole) prophylaxis was administered based on clinicians’ decision, usually until achievement of a stable neutrophil count recovery; during posaconazole administration, VEN was reduced to 100 mg/d as suggested [21]. Antibacterial prophylaxis was generally limited to the outpatient setting and mainly consisted of levofloxacin 500 mg/d orally; inpatients received antibacterial agents only in case of febrile neutropenia due to high local prevalence of carbapenem-resistant *Enterobacteriaceae*. Post-HSCT patients usually received additional prophylaxis with acyclovir and trimethoprim/sulfamethoxazole. Filgrastim support was administered starting from cycle 1 in case of delayed ANC recovery, usually after disease persistence had been ruled out with a BM aspirate; during subsequent cycles, filgrastim was administered as per clinician’s judgement. Antiemetic prophylaxis was given as per local practice. Tumor lysis syndrome prophylaxis was carried out with allopurinol (or rasburicase, for particularly high-risk inpatients) and by ensuring adequate fluid intake and urine alkalization either by oral or intravenous route. Other supportive measures included red blood cell and platelet transfusions as clinically indicated. Supportive measures and ancillary medications were the same irrespective of the partner drug. Clinical eligibility for HSCT procedure was evaluated according to EBMT/GITMO guidelines [22]. 

### 2.4. Adverse Events Reporting

Adverse events were termed and graded retrospectively according to the Common Terminology Criteria for Adverse Events (CTCAE) version 5.0. [23] Clinical and laboratory data regarding adverse events and hospitalizations were obtained through examination of patients’ files.

### 2.5. Statistical Analysis

Chi-squared and Fisher’s exact tests were used to compare patient groups (as defined by clinical or biological characteristics) for binary outcomes. OS was defined as the time from VEN initiation to last follow up or death from any cause; EFS was defined as the time from VEN initiation to documentation of refractoriness, relapse or death from any cause; DFS was defined as the time from the first documentation of CR after VEN initiation to date of relapse. All time-to-event endpoints (OS, EFS and DFS) were analyzed using Kaplan-Meier curves and stratified by factors of interest using log-rank tests; relevant covariates were combined in multivariable Cox regression models for time-to-event endpoints (OS, and EFS) in order to assess the impact of clinical and biological characteristics on outcomes. The impact of selected categorical covariates on the likelihood of CR achievement was assessed using a logistic regression model. All analyses were performed using IBM SPSS vers. 25 software. 

## 3. Results 

### 3.1. Patients and Treatments

Between March 2018 and December 2020, 47 patients with R/R AML were treated consecutively at our institution with VEN-based regimens. Forty patients (85%) carried a diagnosis of de novo AML, while 7/47 patients (15%) had secondary AML (myelodysplastic syndrome, *n* = 4; therapy-related, *n* = 3). Median age was 56 years (33–74); 20/47 (42%) patients were aged ≥ 60; 5 patients (10%) were aged ≥70 at the time of VEN initiation. Eleven patients (23%) were treated for primary refractory disease; of these, seven received VEN+AZA, three received VEN+DAC and one patient received VEN+LDAC. In the PIF cohort, previous treatments included a single course of intensified induction (idarubicin 12 mg/mq/d for 3 days + cytarabine 2000 mg/mg/d for 4 days) (*n* = 2), combination of standard induction (3 + 7 +/− midostaurin) and HiDAC-based salvage (*n* = 8), and multiple salvage lines including HiDAC and clofarabine-based regimen (*n* = 1). Eleven patients (23%) were treated for relapse after HSCT; all but one patient had received myeloablative conditioning regimens. Three patients had received HSCT with active disease. Two patients had extramedullary relapse. In the post-HSCT cohort, the partner drug for VEN was AZA in nine cases and DAC in two. One patient had previously received donor lymphocyte infusions. Twenty-five patients (54%) were treated for first (*n* = 20) or second (*n* = 5) AML relapse; last CR duration was < 6 months for 13 patients, 6–12 months for 9 patients, and >12 months for 3 patients. First- and second-line chemotherapy (induction, consolidation and reinduction regimens) were quite homogenous, the main difference being the use of an intensified induction regimen (as stated above) in selected, younger patients (*n* = 7). FLT3-ITD or TKD-positive patients received midostaurin-containing regimens. One FLT3-ITD positive patient received gilteritinib in first relapse prior to treatment with venetoclax after disease progression. Partner drug was AZA for 13 patients and LDAC for 12 patients. Differences in the choice of partner drug between the three different settings were mainly attributable to off-label prescription limitations based on local policy. Globally, 24 patients (51%) were treated on an intention-to-transplant (ITT) basis; median age was significantly lower in the ITT group (54 years; range 33–66) compared to the non-ITT group (57 years, range 37–74; *p* = 0.041); patients >70 years were all allocated in the non-ITT group. Patients’ and treatment details are summarized in Table 1.

### 3.2. Biological Characteristics

Cytogenetic and molecular data were available for all 47 patients, and they are summarized in Table 1. All patients had NPM1 and FLT3 mutational screening available at baseline. As anticipated, cytogenetic and molecular characteristics were unbalanced between the three different settings: specifically, high risk cytogenetics were significantly more common (*p* = 0.026) in the post-HSCT (4/11, 36%) and PIF (5/11, 45%) groups compared to the relapsed group (2/25, 8%); moreover, patients from the relapsed population were much more likely to bear a NPM1 mutation (13/25, 52%) compared to the post-HSCT (1/11, 9%) and PIF populations (0/11) (*p* = 0.002). Differences in the distribution of FLT3-ITD mutations did not reach statistical significance. Other features reported in Table 1 did not differ significantly between the ITT and non-ITT populations. In order to allow clinically meaningful comparisons, we defined three molecular patient groups: NPM1^mut^, FLT3-ITD^neg^ (group 1), NPM1^mut^, FLT3-ITD^pos^ (group 2) and NPM1^wt^, FLT3^any^ (group 3). 

### 3.3. Treatment Details

Overall, 140 VEN-based cycles were administered. Thirteen patients started treatment as inpatients, with 13 overall cycles administered while being hospitalized. All patients received at least one cycle; median cycle number was two (1–24). At the time of data cutoff, 41 patients (87%) had discontinued treatment. Reasons for treatment discontinuation were disease progression/refractoriness (*n* = 26; 64%), unacceptable toxicity (due to prolonged pancytopenia, *n* = 1; 2%), HSCT (*n* = 13; 32%) or death unrelated to disease progression (*n* = 1; 2%). Thirty patients (64%) received posaconazole, usually until CR and stable peripheral counts recovery had been reached. 

### 3.4. Outcomes

The overall composite CR (CCR) rate was 55% (26/47); half of responders (13/26) obtained a CRi/CRp status. Of all patients in the CCR group, 16 patients (61%) achieved an MRD negative status evaluated by flow cytometry or RT-qPCR. All CRs invariably occurred during the first six weeks of treatment. MRD negativity status was invariably achieved by cycle 2 in the case of patients undergoing flow cytometry-based monitoring and by cycle 3 in NPM1-mutated patients undergoing RT-qPCR-based monitoring. No cases of molecular MRD negativity were observed among CBFB/MYH11 and RUNX1/RUNX1T1 positive patients. CR rates differed significantly between the three different settings (relapsed: 18/25, 72%; PIF: 6/11, 54%; post-HSCT: 2/11, 18%; *p* = 0.02) and CR rate in HSCT-naïve patients was 66% (24/36). Conversely, NPM1 and FLT3 mutational status, the presence of high-risk cytogenetic features, ELN 2017 risk category and the choice of partner drug for VEN did not have any impact on the probability of CR achievement. Age > 60 was shown to impact favorably on the CR rate (age > 60: 15/20, 75%; age < 60: 11/27, 41%; *p* = 0.017). In multivariate analysis, both age > 60 and previous-HSCT independently affected the probability of CR achievement (odds ratio, 1.87; 0.26; *p* = 0.005; *p* = 0.003). Peripheral blast clearance was measured in 14 patients on day 4 after therapy initiation; median PBC did not differ significantly between responders (median: −1.90 logs; −4 to +0.91 logs) and refractory patients (median: −1.56 logs; −3.78 to +0.45 logs), *p* = 0.39.

DFS for the entire cohort of CR patients (*n* = 26) was 10.6 months. ELN 2017 risk category was the only factor yielding a statistically significant impact on DFS duration: ELN favorable, median not reached; ELN intermediate, 10.6 months; ELN adverse, 2 months (*p* = 0.049). Even if not statistically significant, a trend effect on DFS was documented for NPM1/FLT3 molecular risk categories: in group 1, median DFS was not reached, as compared to 4.5 months for group 2 and 11.5 month for group 3; *p* = 0.26) and subsequent HSCT post venetoclax-induced CR (HSCT, median not reached; non-HSCT, 8.4 months; *p* = 0.18). 

EFS for the entire cohort (*n* = 47) was 4.5 months. No impact on EFS was documented for high-risk cytogenetics and ELN 2017 risk group. Interestingly, the rate of early EFS events reflecting disease refractoriness was independent of high-risk cytogenetic features. Conversely, molecular risk group impacted EFS duration in a statistically significant manner (group 1, median not reached; group 2, 1.0 month; group 3, 6.4 months, *p* = 0.003). Notably, FLT3-ITD status by itself retained a significant impact on median EFS (ITD positive, 2.6 months; ITD negative, 6.2 months; *p* = 0.033). In multivariate analysis, FLT3-ITD positivity was the only factor independently affecting EFS (*p* = 0.045). 

**Figure 1:**
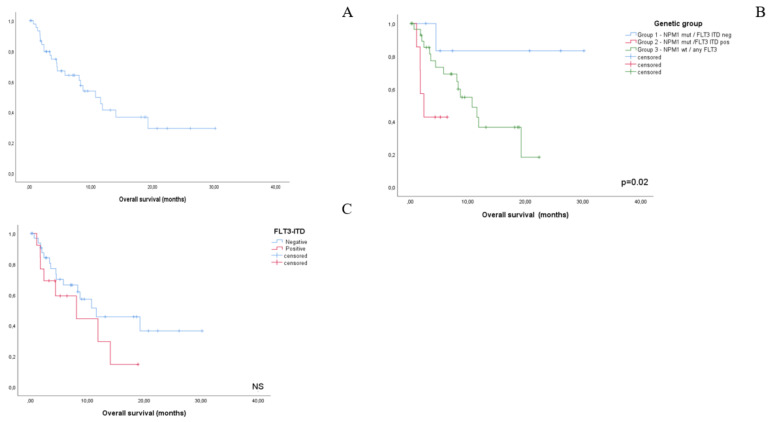
Kaplan-Meier plots representing overall survival for the whole study cohort (**A**), by NPM1/FLT3-ITD-based genetic grup (**B**) and by FLT3-ITD status alone (**C**). Despite not reaching statistical significance, a clear tendency towards a worse outcome for FLT3-ITD positive patients can be noted.

**Figure 2:**
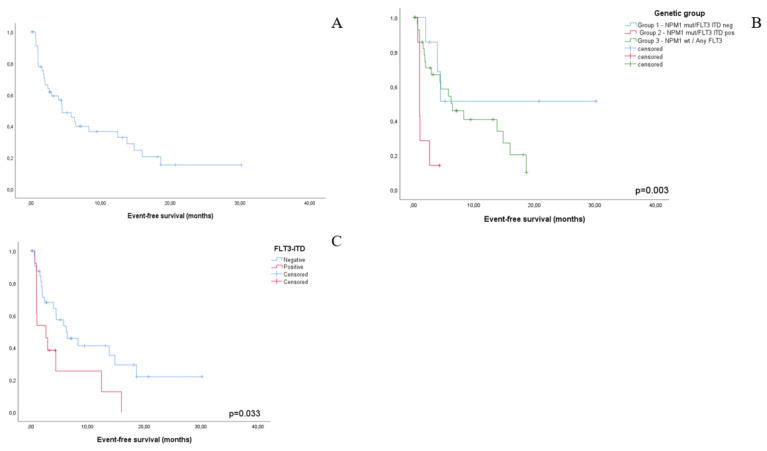
Kaplan-Meier plots representing event-free survival for the whole study cohort (**A**), by NPM1/FLT3-ITD-based genetic group (**B**) and by FLT3-ITD status alone (**C**).

**Figure 3:**
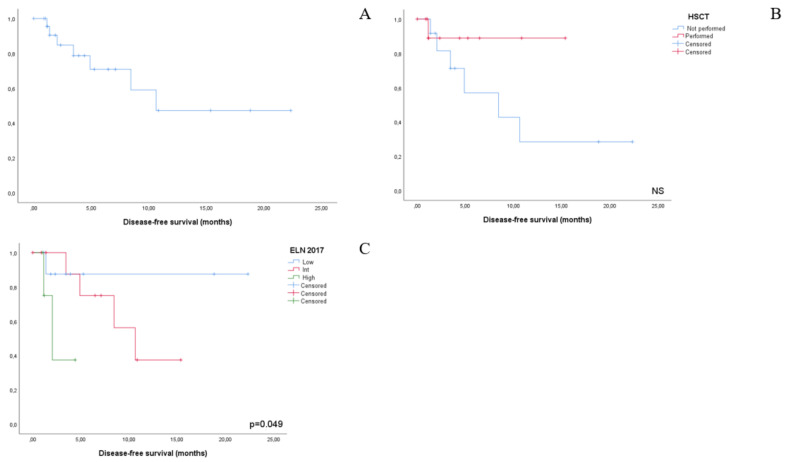
Kaplan-Meier plots representing disease-free survival for the shole study cohort (**A**), by HSCT bridging (**B**) and by ELN 2017 risk group (**C**). Despite not reaching statistical significance due to the small sample size involved, a protective effect for HSCT can be desumed from (**B**).

Overall survival was 10.7 months. ELN 2017 risk category and high-risk cytogenetics did not show an impact on OS duration. Conversely, the molecular risk group significantly impacted OS (group 1, median not reached; group 2, 2.3 months; group 3, 10.7 months; *p* = 0.02). FLT3-ITD positivity associated with shortened, yet not statistically significant, OS (8.2 months vs 11.5 months, NS) (Figure 1, Figure 2 and Figure 3). Efficacy outcomes are summarized in Table 2.

In the ITT group, 13/24 (54%) patients were successfully bridged to HSCT; failure to proceed to HSCT was due exclusively to disease refractoriness. The median number of cycles before HSCT was two; two patients received >2 cycles (3 and 4 cycles, respectively) and three patients received only one cycle before HSCT. Eleven patients were in CR at the time of HSCT; two patients had disease relapse and underwent HSCT with active disease. Conditioning regimen was myeloablative for 9/13 patients and reduced-intensity for 4/13 patients. Stem cell sources were haploidentical siblings (*n* = 5), fully matched Adbverunrelated donors (*n* = 5), fully matched siblings (*n* = 1) and partially matched unrelated donor (*n* = 1). At the time of data cutoff, five patients in the HSCT group had died: four patients died from transplant-related infectious complications and one patient died from severe acute GVHD. 

At the time of data cutoff, 23/47 patients were still alive; median follow-up for the entire cohort was 10.7 months (0.8–30).

### 3.5. Adverse Events

Overall, 21 febrile neutropenia events without know etiology were noted; additionally, 17 microbiologically defined infectious events were recorded (G2, *n* = 10; G3, *n* = 4; G4, *n* = 2). Ten patients experienced oral mucositis, which was graded >2 in only one case. Myelosuppression was the most commonly occurring AE. Cytopenias were evaluated and graded as adverse events only in responding patients and were deemed largely attributable to AML in refractory patients. The majority of patients experienced grade 4 neutropenia (47/47, 100%) and transfusion-dependent anemia and thrombocytopenia (45/47, 95%).

Dose reductions due to cytopenias were needed in 35/47 patients (74%); ultimately, 12 patients were able to tolerate dose level 1 continuously, 31 patients required a dose reduction to level 2, and 4 patients required further dose reduction to level 3. Dose level 2 has gradually become our dosing scheme of choice for R/R AML patients. 

One patient died from intracranial hemorrhage, which was not considered directly attributable to treatment with venetoclax. No clinical/laboratory TLS events were noted. No relevant organ-specific toxicities were documented. AEs are summarized in Table 3.

## 4. Discussion

Relapsed and refractory disease is a common scenario in AML; there is no standard salvage regimen for R/R AML, and different schemes have shown comparable and overall unsatisfactory results. Here, by using VEN as backbone of treatment, we report an overall CCR rate of 55%—a remarkable proof of efficacy in such a heavily treated patient population. CCR rates differed significantly between different treatment settings; a CCR rate of 72% was achieved in relapsed patients that is by itself definitely surprising taking into account that 22/25 patients (88%) had shown a previous CR duration <12 months. Even if this patient population was enriched with NPM1^mut^ cases, in which VEN is known to exert a potent antileukemic effect, such a high CCR rate has not been reported in relapsed AML patients to date [24]. On the other hand, CCR rate for post-HSCT patients was markedly lower (18%), although at least comparable to other commonly adopted regimens in this setting; a favorable toxicity profile should be underlined [25]. Conversely, the high CCR rate (54%) in PIF patients may represent a proof of concept that venetoclax is capable of overcoming at least some of the mechanisms associated with resistance to conventional chemotherapy. This is reinforced by findings that predictive biomarkers (i.e., NPM1 and FLT3 status; high-risk cytogenetics) commonly associated with chemosensitive/chemorefractory disease did not show a significant impact on CCR rate in this study population. Unexpectedly, age >60 was positively associated with higher CCR probability; we surmise that this finding may be largely ascribed to that fact that the age >60y group was enriched for NPM1-mutated (7/20 vs. 7/27, NS) and HSCT-naïve patients (0/20 vs. 11/27, *p* = 0.001). 

Clinical response to VEN occurred almost invariably after a single treatment course, similar to intensive salvage chemotherapy and different from other currently available targeted agents, such as enasidenib, ivosidenib, gilteritinib that usually necessitate several cycles of treatment before inducing measurable responses. At this regard, we evaluated the relationships between early peripheral blast clearance and clinical response. The fact that the magnitude of peripheral blast clearance during the first three days of treatment did not differ between responding and refractory patients is in contrast with our previous findings in a conventional induction setting [26] and remains to be clarified. 

Clinical outcomes differed significantly according to genetic characteristics. Patients with a NPM1^mut^/FLT3-ITD^neg^ (Group 1) molecular profile fared particularly well with regard to all outcome measures; this is in line with previous reports, which, however, are mostly derived from the frontline setting [27]. Conversely, a NPM1^mut^/FLT3-ITD^pos^ (group 2) genotype was associated with a particularly dismal outcome due to frequent VEN resistance or very early relapse. Even when considering FLT3-ITD status alone, a statistically significant detrimental effect on EFS was noted, reflecting a high rate of early treatment failures, in line with previous reports [28]. The relatively low number of FLT3-mutated patients in our cohort mandates caution in interpreting our observations; nonetheless, it is probably advisable that FLT3-ITD^pos^ patients preferentially be managed with either approved or experimental treatments including FLT3-inhibitors. Combining venetoclax with FLT3 inhibitors (i.e., gilteritinib) represents an attractive approach and is currently being evaluated in clinical studies (NCT04140487; NCT03625505; NCT03661307) with encouraging preliminary results (Daver et al., ASH 2020 oral abstract). Patients bearing an NPM1^wt^/FLT3^any^ genotype (Group 3) had an intermediate outcome: this patient group is clearly heterogeneous and will require further characterization from a biological point of view in order to identify novel predictive biomarkers of sensitivity to venetoclax. 

Although more than 60% of CCR patients achieved an MRD negative status, responses were short-lived, and as a consequence, early HSCT is required for long-term survival. Notably, in the ITT population included in the current study, the HSCT bridging rate was remarkable, and this goal was obtained with a notably favorable safety profile, as supported by the fact that the only reason for failure to proceed to HSCT was represented by persistence of disease. Intriguingly, a small fraction of responding patients who did not undergo HSCT are still on treatment and in deep CR after a median of 19 months; this observation—derived from a such a poor risk population—is reminiscent of data reported by DiNardo et al. in elderly patients treated in the frontline setting [27].

The safety profile in our study cohort did not differ from previous data. The most common adverse events were cytopenias which typically improved at response onset, although often incompletely; interestingly, and different from what is commonly observed in the context of intensive chemotherapy, even in MRD-negative responders, incomplete hematological recovery was frequent [29]. However, treatment discontinuation due to excessive myelosuppression occurred in only one patient. No significant end-organ damage events were noted. We believe these findings have remarkable clinical impact, considering that most patients carried on a heavy toxicity burden from previous treatments. 

Despite the retrospective nature of this study that represents its main limitation, current data supports the efficacy and safety of VEN-based regimens in R/R AML without FLT3-ITD mutations, questioning the paradigm in which higher efficacy invariably comes with higher toxicity. These findings may have implications for the current management of R/R AML patients and definitely warrant further exploration in a prospective setting.

## Figures and Tables

**Table 1:** Patients’ characteristics.

Parameter	*n* (%)
Patient number	47
Sex	
Male	26 (55.3%)
Female	21 (44.7%)
Median Age (range)	56 (33–74)
Age ≥ 60	20 (42.5%)
Age ≥ 70	5 (10%)
De novo AML	40 (85%)
Secondary AML	7 (15%) MDS, *n* = 4 Therapy-related, *n* = 3
**ELN 2017 risk at diagnosis**	
Favorable	16 (34%)
Intermediate	17 (36%)
Adverse	7 (15%)
NA	7 (15%)
**NPM1/FLT3 status**	
NPM1^mut^ FLT3-ITD^wt^	7 (15%)
NPM1^mut^ FLT3-ITD^pos^	6 (13%)
NPM1^neg^ FLT3-ITD^wt^	27 (57%)
NPM1^neg^ FLT3-ITD^pos^	7 (15%)
**Cytogenetic risk**	
Low risk	5 (11%)
Intermediate risk	31 (66%)
Poor risk	8 (17%)
Unknow	13 (28%)
**Setting**	
First relapse	20 (43%)
Subsequent relapse	5 (11%)
Relapse after HSCT	11 (23%)
**Partner drug**	
Azacitidine	29 (62%)
Decitabine	5 (11%)
Low-dose cytarabine	13 (28%)
Median cycle number (range)	2 (1–24)
**Intention to treat**	
Intention to HSCT	24 (51%)
Nointention to HSCT	23 (49%)
HSCT performed	13/24 (54%)

**Table 2:** Outcomes.

	Total Patients (*n* = 47)	NPM1^mut^ FLT3-ITD^neg^ (*n* = 7)	NPM1^mut^ FLT3-ITD^pos^ (*n* = 6)	NPM^wt^ FLT3^any^ (*n* = 34)
CCR—no. (%) CRi/CRp—no. (%)	26 (55%) 13 (50% of all CCR patients)			
SD—no. (%)	6 (12%)			
PD—no. (%)	15 (32%)			
DSF mo (median)	10.6	NR	(4.5)	(11.5)
EFS mo (median)	4.5	NR	(1)	(6.4)
OS mo (median)	10.7	NR	(2.03)	10.7

**Table 3:** Adverse events.

Adverse Events	*n* (%)
• Thrombocytopenia ≥ G4	45 (95.7%)
• Neutropenia ≥ G4	47 (100%)
• Febrile neutropenia	21 (45%)
• Anemia ≥ G3	45 (95.7%)
• Infections	17 (36%)
• Mucositis	10 (21%)
• Tumor lysis syndrome	0
